# Outstanding Symptoms of Poststroke Depression during the Acute Phase of Stroke

**DOI:** 10.1371/journal.pone.0163038

**Published:** 2016-10-05

**Authors:** Taizen Nakase, Maiko Tobisawa, Masahiro Sasaki, Akifumi Suzuki

**Affiliations:** Department of Stroke Science, Research Institute for Brain and Blood Vessels–Akita, Akita, Japan; Chiba Daigaku, JAPAN

## Abstract

Poststroke depression (PSD) is a critical complication which might lead to unfavorable outcomes. However, most cases of PSD in the acute phase, during the 2 or 3 weeks following a stroke, are neglected because of the variable comorbid conditions. In this study, aimed at revealing the outstanding symptoms of PSD during the acute phase, consecutive patients with intracranial hemorrhage (ICH) or brain infarction (BI) were asked to fill out a depression questionnaire (Quick Inventory of Depressive Symptomatology Self-Report: QIDS-SR) at 1 week and 1 month following stroke onset. Patients with disturbed consciousness or aphasia were excluded from this study. Forty-nine ICH patients and 222 BI patients completed the QIDS-SR at 1 week and 27 of ICH and 62 of BI at 1 month. The PSD rate was 67% and 46% at 1 week in ICH and BI, respectively. Although sleep disturbance was the most frequent symptom of PSD, psychomotor agitation and appetite disturbance were the most distinguishing symptoms in ICH at 1 week and fatigue at 1 month. On the other hand, most of the depressive symptoms addressed in QIDS-SR were observed in PSD of BI patients both at 1 week and 1 month. In conclusion, while sleep disturbance was a frequent but non-specific symptom, appetite disturbance and fatigue might be critical symptoms to suggest PSD during the acute phase of stroke.

## Introduction

Poststroke depression (PSD) is a critical complication which might lead to an unfavorable stroke outcomes [1–3]. It has been reported that insomnia, apathy, mood disorder and cognitive impairment are major symptoms of PSD [4, 5]. The frequency of PSD has been reported to peak at around 3 to 6 months and 2 to 3 years following the stroke [6–8]. Because PSD in the acute phase can be a hindrance to rehabilitation [9], it can be argued that interventions aimed at addressing PSD in the acute phase are as important as the medical treatment for stroke itself. However, PSD during the acute phase of stroke is sometimes difficult to diagnose because of the complicated clinical setting, including impaired consciousness, aphasia, cognitive decline, unstable general condition and the effect of medications [1, 2].

There are many instruments for assessing depression, and each instrument has its clinical utility. The Quick Inventory of Depressive Symptomatology Self-Report (QIDS-SR) was created to assess depressive symptom severity [10]. The results of QIDS-SR have been reported to match the Hamilton Rating Scale for Depression and be sensitive to symptom change [10, 11]. Moreover, QIDS-SR is a simple self-reporting instrument, which means that the examiner does not require training and the patient will bear less load [10, 12].

In this study, acute stroke patients including transient ischemic attack (TIA) were prospectively assessed with the QIDS-SR depression questionnaire to investigate the symptoms which may indicate the possibility of PSD in in-hospital patients.

## Methods

### Patients

This prospective observational study was approved by the ethical committee of the Research Institute for Brain and Blood Vessels (#837). Between May 2013 and April 2014, acute stroke patients who were admitted to our hospital within 48 hours of symptom-onset were consecutively screened. Inclusion criteria of this study were patients with 1) any neurologic deficits and 2) any stroke lesion observed by brain computed tomography (CT) or magnetic resonance image (MRI) on admission. Because this study used self-report questioner sheet, patients showing disturbed consciousness (Glasgow Coma Scale, less than 14: E3 V5 M6) or patients with aphasia were excluded from this study. All enrolled patients were consented with a written document and asked to fill out the depression scale sheet QIDS-SR at 1 week and 1 month following stroke onset. Patients who could not write because of paresis were aided by a helper.

### Classification

Stroke lesion was confirmed by brain CT or MRI. Distribution of the hematoma lesion for intracranial hemorrhage (ICH) was classified into the deep cerebral region (Deep: including basal ganglia, thalamus and putamen), the lobar region (Lobar: including cerebral cortex and white matter) and the infratentorial region (Infra: including brainstem and cerebellum). Distribution of the lesion for brain infarction (BI) was classified into the deep cerebral region (Deep: including basal ganglia, thalamus and deep white matter), the cortical region (Cortical: including cerebral cortex), and the infratentorial region (Infra: including brainstem and cerebellum). The etiology of BI was classified into cardiogenic embolism (CE), atherothrombotic infarction (AT), lacunar infarction (LI) and other type stroke, based on the clinical records. Neurologic severity was assessed by the National Institutes of Health Stroke Scale (NIHSS) score on admission. Data on the clinical backgrounds of all patients were collected from the clinical records. Risk factors were defined as follows: hypertension (>140 mmHg systolic or >90 mmHg diastolic, or currently prescribed anti-hypertensive medication), diabetes mellitus (DM: random blood sugar level >200 mg/dL or currently prescribed anti-diabetic medication), dyslipidemia (>140 mg/dL serum low-density lipoprotein or >150 mg/dL triglyceride, or currently prescribed anti-hyperlipidemia medication), excessive ethanol intake (>30 mL of converted alcohol amount per day) and smoking.

### Treatment

During the acute phase, all patients were treated with standard medications following the Japanese guidelines [13]. If a patient complained of insomnia, a doctor prescribed a sleeping pill. Otherwise, patients had neither been examined by a psychologist nor treated with any psychotropic medicines during the study period. The corresponding doctor of each patient was blinded to the results of QIDS-SR.

### Depression assessment

The QIDS-SR was applied to assess depressive symptoms in our patients. Following the instructions of the QIDS-SR (www.ids-qids.org) and the recommendation of a previous report [10], the presence and severity of depression was classified into no depression (lower than 5), mild (6 to 10), moderate (11 to 15), severe (16 to 20) and very severe (more than 21) depression. Individual domains of the QIDS-SR were categorized into 1) sleep disturbance, 2) sad mood, 3) appetite disturbance, 4) concentration, 5) self criticism, 6) suicidal ideation, 7) interest disturbance, 8) fatigue and 9) psychomotor agitation. Full list of QIDS-SR questions is presented as an appendix (S1 Table).

### Statistical analysis

All data are presented as means ± standard deviations (SD) for continuous variables and percentages (%) for categorical variables. Clinical characteristics and depression symptoms were compared in PSD patients and patients without depression by the Mann-Whitney’s U test for continuous variables and by the Pearson χ^2^ test for categorical variables. Multiple logistic regression analysis was used to investigate the critical factors and domains of QIDS-SR which might affect the presence of PSD. All statistical analysis was performed by JMP9 software (SAS Institute Inc., NC).

## Results

### Patient characteristics

**Fig 1:**
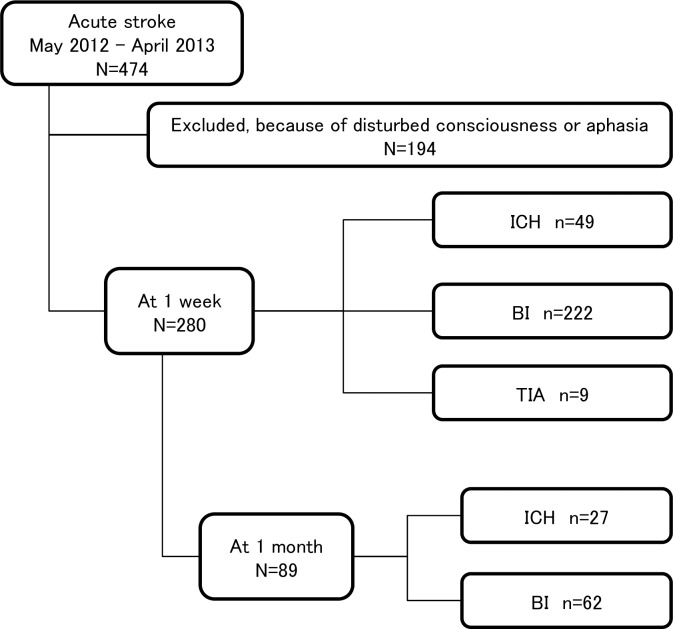
Patients screening flow chart. The selection criteria and number of patients are described.

Of the 474 acute stroke patients, the QIDS-SR was completed by 280 patients (59.1%) at 1 week and 89 (18.8%) at 1 month following stroke onset (Fig 1). Because patients with mild neurologic deficits were discharged within 1 month, the number of in-hospital patients declined significantly at 1 month. The clinical characteristics of ICH patients are shown in Table 1. Most of hematoma lesions were in the deep cerebrum (65%), followed by the infratentorial and lobar regions. Patients of infratentorial hemorrhage were significantly older compared with those of deep cerebral hemorrhage (p = 0.02). The clinical characteristics of BI patients are shown in Table 2. Patients of cardioembolic stroke were significantly older (p = 0.002) and patients of other infarction types were significantly younger than the other groups (p = 0.005). Number of TIA patients was 9, and average age was 62.2±13.1 years. Percentage of males was 77.7%. All TIA patients were discharged before 1 month.

**Table 1:** Clinical characteristics of ICH patients.

	All	Lobar	Deep	Infra
**N**	49	6	32	11
**Sex (female %)**	42.9	66.7	37.5	45.5
**Age (mean±SD)**	64.8±11.9	66.7±18.8	62.1±11.0	71.7±7.6 ^♭^
**Hypertension, n (%)**	45 (91.8)	6 (100)	31 (96.9)	8 (72.7)
**Hyperlipidemia, n (%)**	19 (42.2)	2 (33.3)	10 (31.3)	7 (63.6)
**Diabetes, n (%)**	13 (26.5)	1 (16.7)	8 (25.0)	4 (36.4)
**AF, n (%)**	1 (2.0)	0 (0)	0 (0)	1 (9.1)
**Smoking, n (%)**	20 (40.8)	2 (33.3)	15 (46.9)	3 (27.3)
**Alcohol, n (%)**	15 (30.6)	2 (33.3)	10 (31.3)	3 (27.3)
**Previous stroke, n (%)**	13 (26.5)	3 (50.0)	5 (15.6)	5 (45.5)

AF: atrial fibrillation.

♭: p = 0.021, vs. Deep group.

**Table 2:** Clinical characteristics of BI patients.

	All	CE	AT	LI	others
**N**	222	70	60	83	9
**Sex (female %)**	36.0	42.9	26.7	38.6	22.2
**Age (mean±SD)**	70.4±10.8	74.4±9.9 ^†^	69.2±9.9	69.2±10.5	57.3±11.1 ^‡^
**Hypertension, n (%)**	164 (73.9)	49 (70.0)	51 (85.0)	61 (73.5)	4 (44.4)
**Hyperlipidemia, n (%)**	93 (41.9)	29 (41.4)	28 (46.7)	32 (38.6)	4 (44.4)
**Diabetes, n (%)**	58 (26.1)	15 (21.4)	15 (25.0)	28 (33.7)	0 (0)
**AF, n (%)**	48 (21.6)	29 (41.4)	11 (18.3)	8 (9.6)	0 (0)
**Smoking, n (%)**	116 (52.3)	29 (41.4)	38 (63.3)	43 (51.8)	6 (66.7)
**Alcohol, n (%)**	49 (22.1)	16 (22.9)	14 (23.3)	15 (18.1)	4 (44.4)
**Previous stroke, n (%)**	70 (31.5)	23 (32.9)	20 (33.3)	25 (30.1)	2 (22.2)
**Lesions**					
**Cortical, n (%)**	101 (45.5)	56 (80.0)	43 (71.7)	0 (0)	2 (22.2)
**Deep, n (%)**	76 (34.2)	3 (4.3)	5 (8.3)	68 (81.9)	0 (0)
**Infra., n (%)**	45 (20.3)	11 (15.7)	12 (20.0)	15 (18.1)	7 (77.8)

CE: cardiogenic embolism, AT: atherothrombotic infarction, LI: lacunar infarction, others: other type stroke, AF: atrial fibrillation, Infra.: infratentorial.

†: p = 0.002, vs. AT, LI and others.

‡: p = 0.005, vs. LI.

### Frequency of PSD

**Fig 2:**
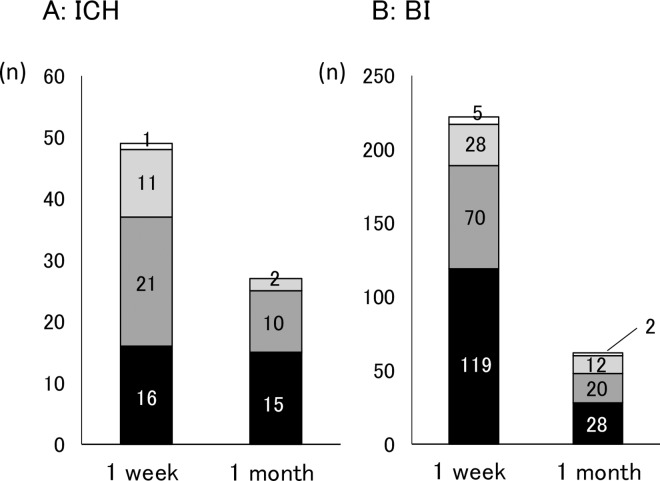
The number of PSD patients at 1 week and at 1 month of ICH (A) and BI (B). At 1 week, 16 of 49 ICH patients (32.7%) expressed no depression. Whereas, 119 of 222 BI patients (53.6%) showed no PSD. The number of patients with PSD among ICH patients was decreased at 1 month. The distribution of the severity of depression was mostly consistent among all PSD patients. Black, dark gray, light gray and white indicate no depression, mild depression, moderate depression and severe depression, respectively. The numbers in the columns are the number of patients.

PSD was observed 48.6% and 51.7% of in-hospital patients at 1 week and 1 month following the onset, respectively. When assessing ICH and BI patients separately (Tables 3 and 4), the rates in ICH were 63.3% and 44.4% at 1 week and 1 month, respectively, whereas the rates in BI were 46.4% and 54.8% at 1 week and 1 month, respectively. The numbers of ICH patients who were assessed PSD or no depression were shown in stacked graphs (Fig 2A). The distributions of the severity of depression at 1 week were 63.6%, 33.3% and 3.1% for mild, moderate and severe depression, respectively. Furthermore, at 1 month, the rates of mild depression and moderate depression were 83.3% and 16.7%, respectively. The numbers of BI patients who were assessed PSD or no depression were shown in stacked graphs (Fig 2B). The distributions of the severity of depression at 1 week were 67.4%, 28.3% and 4.3% for mild, moderate and severe depression, respectively. The distribution of the depression severity was similar at 1 month. Only 22% of in-hospital TIA patients showed mild PSD at 1 week. There was no difference of the PSD incidence between males and females either at 1 week or 1 month (data not shown).

**Table 3:** Depressive domains in PSD of ICH patients.

	At 1 week			At 1 month		
Domain	without depression	PSD	P	without depression	PSD	P
**N (%)**	18 (36.7)	31 (63.3)		15 (55.6)	12 (44.4)	
**Sleep disturbance**	15 (83.3)	31 (100)	0.082	14 (93.3)	12 (100)	0.999	
**Sad mood**	4 (22.2)	18 (58.1)	0.032	2 (13.3)	4 (33.3)	0.427	
**Appetite disturbance**	7 (38.9)	27 (87.1)	0.001	11 (73.3)	8 (66.7)	0.999	
**Concentration**	4 (22.2)	15 (48.4)	0.130	0 (0)	6 (50.0)	0.008	
**Self criticism**	7 (38.9)	20 (64.5)	0.148	3 (20.0)	5 (41.7)	0.414	
**Suicide ideation**	1 (5.6)	9 (29.0)	0.108	0 (0)	1 (8.3)	0.879	
**Interest disturbance**	3 (16.7)	11 (35.5)	0.278	1 (6.7)	1 (8.3)	0.999	
**Fatigue**	5 (27.8)	11 (35.5)	0.806	4 (26.7)	9 (75.0)	0.034	
**Psychomotor agitation**	2 (11.1)	24 (77.4)	<0.001	4 (26.7)	8 (66.7)	0.089	

**Table 4:** Depressive domains in PSD of BI patients.

	At 1 week			At 1 month		
Domain	without depression	PSD	P	without depression	PSD	P
**N (%)**	119 (53.6)	103 (46.4)		28 (45.2)	34 (54.8)	
**Sleep disturbance**	103 (86.6)	101 (98.1)	0.004	24 (85.7)	34 (100)	0.079
**Sad mood**	28 (23.5)	59 (57.3)	<0.001	3 (10.7)	19 (55.9)	0.001
**Appetite disturbance**	47 (39.5)	76 (73.8)	<0.001	15 (53.6)	26 (76.5)	0.104
**Concentration**	14 (11.8)	47 (45.6)	<0.001	4 (14.3)	18 (52.9)	0.004
**Self criticism**	16 (13.4)	76 (73.8)	<0.001	3 (10.7)	19 (55.9)	0.001
**Suicide ideation**	3 (2.5)	27 (26.2)	<0.001	1 (3.6)	9 (26.5)	0.036
**Interest disturbance**	5 (4.2)	33 (32.0)	<0.001	1 (3.6)	17 (50.0)	<0.001
**Fatigue**	35 (29.4)	83 (80.6)	<0.001	7 (25.0)	21 (61.8)	0.008
**Psychomotor agitation**	34 (28.6)	22 (21.4)	0.281	6 (21.4)	24 (70.6)	<0.001

### Stroke features and PSD

**Fig 3:**
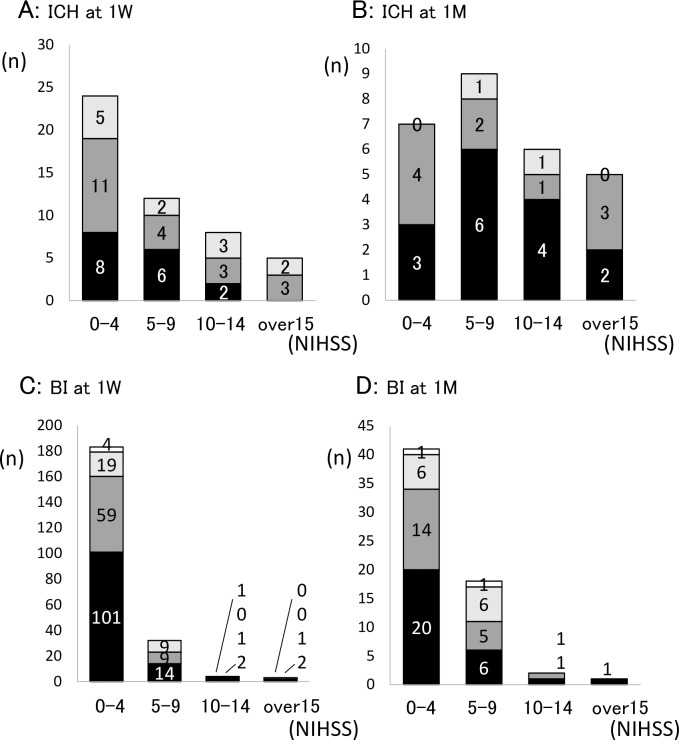
The number of PSD patients classified by the neurological severity. There is no relation between the incidence of PSD and the severity of neurological deficits in either 1 week or 1 month in ICH or BI patients (A, B, C and D, respectively). National Institute of Health Stroke Scale (NIHSS) score was classified into 0–4, 5–9, 10–14 and over 15. Black, dark gray, light gray and white indicates no depression, mild depression, moderate depression and severe depression, respectively. The numbers in the columns are the number of patients.

**Fig 4:**
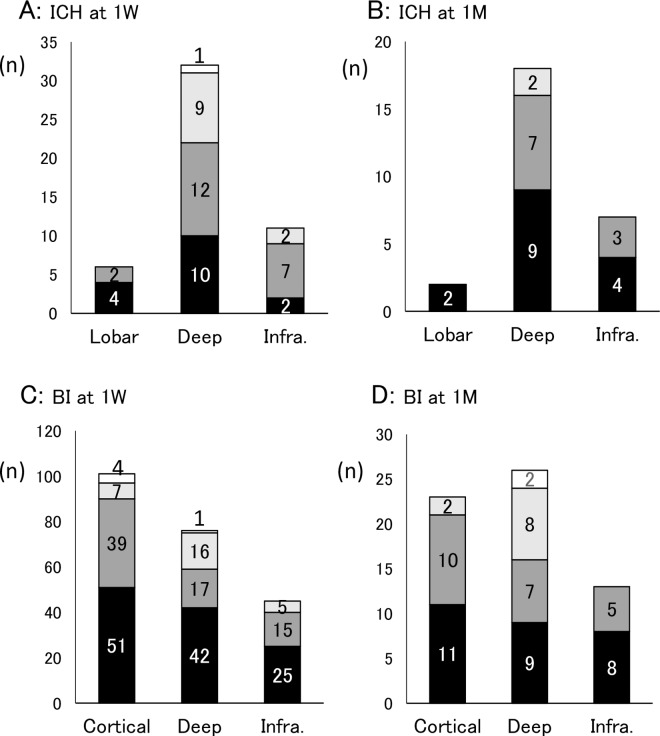
The number of the PSD patients classified by the lesion location in ICH (A and B) and BI (C and D). The number of PSD patients was higher in deep and infratentorial hemorrhage compared with lobar hemorrhage at both 1 week and 1 month, although there is no statistical difference (A and B). There is no difference of PSD frequency among different ischemic lesion at 1 week (C). The number of depressive patients was smaller in infratentorial ischemia compared with cortical and deep ischemic lesions at 1 month (D). Black, dark gray, light gray and white indicate no depression, mild depression, moderate depression and severe depression, respectively. The numbers in the columns are the number of patients.

There was no significant relationship between stroke severity and the incidence of PSD either in ICH or BI patients (Fig 3). Regarding lesion distribution in ICH patients, deep and infratentorial hemorrhages showed higher number of PSD patients compared with lobar hemorrhage (68.7%, 81.2% and 33.4%, respectively) at 1 week (Fig 4A), although there was no statistically significant difference (p = 0.056). At 1 month, these trends were still observed (Fig 4B). On the other hand, the frequency of PSD patients in BI was similar among cortical, deep and infratentorial lesions at 1 week (Fig 4C), but the ratio of PSD patients in deep lesion was higher compared with patients without depression at 1 month (p = 0.078) (Fig 4D). There was no significant difference in the PSD incidence between right and left hemispheric lesions (data not shown).

### PSD symptoms

The most frequent symptom of the depressive domain was sleep disturbance in both ICH and BI (Tables 3 and 4). However, patients without depression also reported sleep disturbance in high frequency. In ICH (Table 3), appetite disturbance and psychomotor agitation were exhibited in a significantly higher percentages of PSD patients at 1 week compared with patients without depression (p = 0.001 and p<0.001, respectively). At 1 month, the frequency of fatigue became significantly higher in PSD patients (p = 0.03). In BI (Table 4), most of the domains of depression, except for psychomotor agitation, were exhibited in significantly high frequencies by PSD patients compared with patients without depression at 1 week (p≤0.004). At 1 month, the frequency of most domains, except for sleep disturbance and appetite disturbance, were still significantly higher in PSD patients compared with patients without depression (p≤0.036).

Odds ratio (OR) was calculated for each clinical factors and QIDS-SR domains of which significant difference was observed by χ^2^ test using multiple logistic regression analysis in ICH patients (Table 5) and BI patients (Table 6). At 1 week in ICH patients, sad mood and psychomotor agitation could still be significant risks of having PSD (OR: 15.78 and 14.16, p = 0.004 and 0.007, respectively). At 1 month, fatigue was a significant risk of having PSD (OR: 12.16, p = 0.027). At 1 week in BI patients, all selected domains of QIDS-SR were significant risks of having PSD. At 1 month, all selected domains were also significant risks of having PSD.

**Table 5:** Odds ratio of the QIDS-SR domains in PSD of ICH patients.

	At 1 week			At 1 month		
Domain	Odds ratio	95% CI	P	Odds ratio	95% CI	P
**Sad mood**	15.78	2.47–100.91	0.004			
**Appetite disturbance**	9.53	0.88–102.74	0.063			
**Concentration**				>1000	0-∞	0.970
**Fatigue**				12.16	1.33–111.48	0.027
**Psychomotor agitation**	14.16	2.08–96.40	0.007			

**Table 6:** Odds ratio of the QIDS-SR domains in PSD of BI patients.

	At 1 week			At 1 month		
Domain	Odds ratio	95% CI	P	Odds ratio	95% CI	P
**Sleep disturbance**	6.87	1.52–30.97	0.002			
**Sad mood**	4.21	2.37–7.49	<0.001	10.56	2.67–41.78	<0.001
**Appetite disturbance**	4.53	2.55–8.07	<0.001			
**Concentration**	6.13	3.11–12.07	<0.001	6.75	1.93–23.67	0.002
**Self criticism**	17.30	8.75–34.23	<0.001	10.56	2.67–41.78	<0.001
**Suicide ideation**	21.37	4.95–92.33	<0.001	9.72	1.15–82.32	0.015
**Interest disturbance**	13.84	4.71–40.68	<0.001	27.00	3.29–221.84	<0.001
**Fatigue**	5.82	3.26–10.38	<0.001	4.85	1.61–14.56	0.004
**Psychomotor agitation**				8.80	2.74–28.23	<0.001

## Discussion

Our results demonstrate that PSD in the acute phase of stroke is observed in almost half of acute stroke patients. Sleep disturbance is the most frequent, albeit non-specific, symptom. Appetite disturbance and psychomotor agitation at 1 week and fatigue at 1 moth could be the distinguishing symptoms for acute phase PSD in ICH patients. On the other hand, PSD in BI patients show more various depressive symptoms compared with those in ICH patients.

### Clinical characteristics of PSD

Several studies have examined the relationship between the onset of PSD and the location of the stroke lesion: whereas left hemispheric lesions might relate to PSD observed within 2 months [14–17], late onset PSD was reported to relate to right-sided lesions [17]. However, it should be noted that the timing of PSD assessment was not consistent in previous studies [18], resulting in the difficulty of examining the relationship between PSD onset and the laterality of the stroke lesion [19–21]. Our results showed no laterality bias of stroke lesion and PSD at both 1 week and 1 month following stroke onset. From the viewpoint of stroke subtype, the prevalence rate of PSD is higher at 1 month in patients with lacunar infarction and the other type stroke, although the number of patients in these groups was small. In fact, a lesion in the lenticulocapsular area has been pointed out as a predictor of early depression after stroke [16], and vascular depression has been considered to relate to deep white matter and corticostriate ischemic lesions [22]. Our results empirically support these findings. Moreover, PSD was more frequently observed in the infratentorial region in ICH compared with BI. Because hematoma causes an increase in intracranial pressure, especially at the infratentorial area, this phenomenon might trigger the unfavorable psychological symptoms.

### Clinical features of PSD

Although sleep disturbance was the most frequent symptom, patients without depression also complained of sleep disturbance in both ICH and BI, suggesting that this might be a nonspecific domain of PSD. Meanwhile, at 1 week, ICH patients showed appetite disturbance and psychomotor agitation as PSD symptoms, unlike BI patients who did not exhibit psychomotor agitation at 1 week. At 1 month, fatigue was a significant PSD symptom in ICH patients, and most of symptoms except for appetite disturbance were significant in BI patients. Thus, the pathogenesis of PSD may be different in ICH and BI patients. Nonetheless, it can be postulated that appetite disturbance and fatigue could be two distinguishing symptoms of PSD in both ICH and BI at 1 week and 1 month, respectively. Previous studies reported that immediate treatment of PSD is important for the advancing of rehabilitation [23, 24] as well as for the improvement of long-term outcomes [25]. Therefore, if a patient complained of sleep disturbance accompanied by appetite disturbance or fatigue during the acute phase, a diagnosis of PSD should be considered.

### Study limitations

This study has some potential limitations. First, depressive symptoms of PSD were assessed using only the QIDS-SR. However, it was reported that any of the instruments which were used for the screening of PSD, including Beck Depression Inventory, Hamilton Rating Scale for Depression and Clinical Global Impression, were equally useful in assessing depression [26]. The assessment of QIDS-SR which was adopted for the screening of PSD in this study has been reported to match the results of the Hamilton Rating Scale for Depression, and be a simple self-report instrument, enabling the assessment of compromised patients [10]. Moreover, the QIDS-SR was reported to be a test sensitive to changes of symptom severity in depression [11]. Therefore, although various kinds of assessment tests have been used for the diagnosis of PSD, the QIDS-SR could be reasonably adopted for the assessment of acute stroke patients. However, the number of patients who were followed for 1 month decreased, and the findings could not be simply compared between 1 week and 1 month. Because this study aimed to assess PSD in hospitalized patients, discharged patients before 1 month were not tracked. There is a need to follow-up all patients, if a true change of symptoms is to be properly assessed.

Patients with aphasia were excluded from this study. Our results may underestimate PSD in stroke patients who present speech disturbances significantly more commonly than other stroke patients. It remains difficult to assess the existence of depressive symptoms in patients suffering from aphasia. Patients with aphasia have been reported to show almost the same prevalence of PSD as those without aphasia [27]. Benaim et al. reported that the Aphasic Depression Rating Scale was effective for evaluating PSD in aphasia patients during subacute phase following stroke [28]. Even though, the yes/no battery of questions might be easier for cognitive declined patients, it would be difficult to assess patients with severe aphasia [29]. Moreover, it is reported that patients with non-fluent aphasia had higher depression score compared with those with fluent aphasia [30]. Further evaluation of PSD should be organized in patients with aphasia by using appropriate scales which are properly validated.

Finally, since this study focused on the PSD of in-hospital patients, the number of patients was relatively small, especially those who could be followed until 1 month. A larger prospective study, including both hospitalized and discharged stroke patients, is warranted.

## Conclusions

Half of acute stroke patients who continuously remained inpatients exhibited PSD within a month. Critical symptoms which suggested the existence of PSD were appetite disturbance at a week and fatigue at a month following stroke. Awareness of critical symptoms of PSD can be expected to result in better psychological outcomes for stroke patients.

## Supporting Information

S1 Table Full list of QIDS-SR questions. Scoring is made by adding the highest score on any 1 of the 4 sleep items, the score on item 5, the highest score on any 1 of the appetite/weight items, the score on item 10, the score on item 11, the score on item 12, the score on item 13, the score on item 14 and the highest score on either of the 2 psychomotor items. Total score is used for deciding the severity of depression.
(DOCX)
